# 

**Published:** 1885-10

**Authors:** 


CONTENTS.

Vol. 32 No. 10

| | PAGE. |
|---|---|
| Fruits in Summer | 1 |
| Early Marriages | 2 |
| Hay Fever and its Cure | 3 |
| Present Taste in Decoration | 4 |
| Abominations of the Back Yard | 5 |
| The Best Time for Administering Medicines ? | 5 |
| Chloral Hydrate as a Vesicant | 6 |
| Paper Towels for Surgical Purposes | 7 |
| Apomorphia as an Emetic | 7 |
| In a Circular Issued by the Board of Health | 8 |
| The Rapidity of the Circulation of the Blood | 8 |
| General Grant's Last Days | 10 |
| The Snake Dance | 13 |
| Coca | 14 |
| Restoration of Life | 15 |
| The Care of Horses | 15 |
| The Talk of a Dentist | 16 |
| The Danger of Contagion in Throat Troubles | 17 |
| Queer German Superstitions | 18 |
| The New Era of Womanhood | 19 |

